# Delayed Diagnosis of Germline *GATA2* Deficiency Following Severe Adolescent Iliocaval Thrombosis and Decade‐Long Monocytopenia: Diagnostic and Testing Pitfalls

**DOI:** 10.1155/crig/3130765

**Published:** 2026-11-05

**Authors:** Ahsan Wahab, Moujtaba Zahweh, Michael Stone, John G. Quigley

**Affiliations:** ^1^ Division of Hematology/Oncology Department of Medicine University of Illinois Chicago Chicago Illinois USA duhs.edu.pk; ^2^ Department of Pathology University of Illinois Chicago Chicago Illinois USA uic.edu

**Keywords:** bone marrow transplant, *GATA2*, monocytopenia, myelodysplasia, thrombosis

## Abstract

*GATA2* haploinsufficiency is an autosomal dominant germline bone marrow disorder with heterogeneous manifestations, including cytopenias, recurrent infections, and progression to myelodysplastic syndrome (MDS)/acute myeloid leukemia (AML). Vascular complications, including venous thrombosis, are increasingly recognized. We present a male who developed extensive iliocaval thrombosis at age 16 after minor surgery and had persistent absolute monocytopenia, which remained unaddressed. He subsequently developed recurrent urogenital chlamydial infections and progressive cytopenias. Bone marrow evaluation revealed hypocellularity with dysmegakaryopoiesis and dysgranulopoiesis, consistent with hypocellular MDS; cytogenetics showed a gain of 1q with der(1; 21) (q10; q10). Comprehensive myeloid next‐generation sequencing panel (that included *GATA2*) identified truncating *STAG2*, but no *GATA2* coding mutations. Hereditary bone marrow failure testing of blood and skin fibroblasts, however, revealed a *GATA2* intron 4 enhancer (+9.5Kb) variant, confirming *GATA2* deficiency. He was referred for allogeneic hematopoietic stem cell transplant. During the pretransplant phase, anticoagulation was temporarily held for 3 days for central venous catheter placement, which resulted in renal infarction, indicating his ongoing high risk of thrombosis. This case highlights missed opportunities to diagnose *GATA2* deficiency: the presence of persistent monocytopenia and the development of extensive venous thrombosis in an adolescent following minor surgery. In addition, it demonstrates the limitations of standard somatic exome‐focused (‘myeloid disorder’) panels in detecting noncoding *GATA2* germline variants. In addition, for patients on anticoagulation with a history of extensive thrombosis or considered to be at high risk of recurrent thrombosis, periprocedural bridging should be considered, after careful assessment of thrombotic and bleeding risks. Early recognition of *GATA2* deficiency enables timely genetic confirmation, cascade testing of family members and risk‐adapted strategies to prevent progression to AML, recurrent thrombosis, and infections.

## 1. Introduction

*GATA2* haploinsufficiency is an autosomal dominant germline disorder caused by pathogenic variants in the *GATA2* gene, which encodes GATA binding protein 2, a transcription factor essential for hematopoietic stem cell differentiation [1]. *GATA2* haploinsufficiency is phenotypically heterogeneous, characterized by a range of syndromic entities including MonoMac syndrome (a cardinal phenotype characterized by monocytopenia [Mono] and susceptibility to *Mycobacterium* avium complex [MAC] and viral and fungal infections [2] occurring in late childhood to early adulthood), Emberger syndrome (primary lymphedema associated with leukemia or myelodysplasia), DCML (dendritic cell, monocyte, B and natural killer [NK] lymphoid) deficiency, and a syndrome of familial MDS/AML [1, 3]. These phenotypes involve multiple organs, most commonly affecting the hematologic, immunologic, vascular, and lymphatic systems; for example, unprovoked thrombosis occurs in up to 25% of patients with *GATA2* deficiency [3, 4]. In addition to these known syndromes, new presentations are emerging with increasing recognition of the disorder [5]. Here, we present a unique case of an adolescent with unexplained extensive deep venous thrombosis (DVT) and prolonged absolute monocytopenia persisting for more than a decade who was ultimately diagnosed with germline *GATA2* haploinsufficiency due to a pathogenic intron 4 enhancer point mutation—a relatively uncommon variant [2, 6]. Bone marrow (BM) examination revealed evidence of MDS, prompting timely referral for allogeneic hematopoietic stem cell transplantation (allo‐HSCT). There were multiple missed opportunities for early diagnosis. Moreover, he developed renal infarction when anticoagulation (AC) was temporarily held prior to transplant, suggesting the need for consideration of AC bridging in select patients with a history of extensive DVT when undergoing procedures.

## 2. Case Presentation

A 23‐year‐old man was referred to the adult hematology clinic for AC recommendations in the setting of prior DVT. At age 16, he underwent an uncomplicated repair of a shoulder labral tear without postoperative mobility limitations. Approximately 3 weeks postoperatively, he presented with extensive DVT of the left lower extremity involving the common iliac, external iliac, and common femoral and mid popliteal veins, with extension into the inferior vena cava (IVC) to the level of the renal veins. CT abdomen/pelvis demonstrated focal narrowing of the left iliac vein behind the right common iliac artery, raising suspicion for May–Thurner syndrome. He underwent interventional radiology‐guided thrombolytic therapy and was started on intravenous heparin. His postprocedure course was complicated by a right gastrocnemius hematoma and compartment syndrome, requiring urgent fasciotomy. IVC filter was placed due to AC interruption. Subsequently, he underwent mechanical thrombectomy and stenting of the left iliac vein, restoring venous flow from the femoral vein to the IVC, and started on rivaroxaban.

He had no personal or family history of thrombosis. A hypercoagulable workup at presentation, while receiving low‐molecular‐weight heparin, was negative for evidence of antiphospholipid syndrome (lupus anticoagulant within the normal range, negative for IgG and IgM antibodies to cardiolipin and beta2 glycoprotein), prothrombin G20210A or Factor V Leiden mutations. Antithrombin III, protein C, and protein S activity levels were all normal. He made a rapid recovery; the IVC filter was removed after three months, and rivaroxaban continued.

At age 4, he had surgery for an oral squamous papilloma, positive for HPV 6/11 by in situ hybridization. Beginning at age 16, he developed recurrent urogenital chlamydial infections, with gonococcal co‐infection at age 22, all adequately treated. He had no other medical history of note; he denied a history of mycobacterial or viral infections, deafness, or lymphedema, and his family history was negative for hematologic disorders, including a negative history of thrombosis.

Continuation of rivaroxaban was recommended, and in view of his initial presentation with thrombosis, evidence for PNH and JAK2 gene mutation investigated (both negative). Notably, his complete blood count (CBC) revealed monocytopenia, prompting review of all prior CBCs, which demonstrated a history of severe monocytopenia going back to his first recorded EMR CBC data (2013; age 11), ranging from 0.0 to 0.1 × 10^9^/L (reference range [RR]: 0.3–1.1 × 10^9^/L; see Table 1).

**TABLE 1:** Timeline of monocytopenia and recurrent chlamydial infections pre‐ and posttransplant.

Timeline	Month/year	WBC (3.9–11.0 × 10^9^/L)	ANC (1.5–7.5 × 10^9^/L)	ALC (1.3–3.9 × 10^9^/L)	AMC (0.3–1.1 × 10^9^/L)	Hgb (13.1–17.6 g/dL)	MCV (80–99 fL)	Platelets (150–450 × 10^9^/L)	Urogenital infections
Pretransplant	^∗^February 2013	4.7	1.3	3.2	0.1	13.7	83	240	—
	Dec 2017	4.3	1.9	2.2	0.0	14.9	87.1	152	Chlamydia
	Mar 2019	5.6	1.3	3.0	0.0	16.4	87.5	268	Chlamydia (co‐infection with *N. gonorrhoeae*)
	Oct 2023	3.1	1.6	1.4	0.0	14.7	92.8	278	Chlamydia
	Mar 2025	2.5	1.6	0.8	0.0	11.7	93.9	192	Chlamydia
	Nov 2025	1.6	0.8	0.8	0.0	10.7	95.6	134	—

Transplant	January 2026	**1.0**	**1.0**	**0.0**	**0.0**	**10.4**	**94.1**	**115**	—

Posttransplant	July 2026	4.9	3.0	1.0	**0.5**	9.6	103.1	231	—

*Note:* Transplant values are for day of transplant; they have been made bold to create a visual difference from the rest of the labs. In addition, WBC and monocyte count are bold posttransplant to indicate their recovery to normal values posttransplant.

Abbreviations: ALC, absolute lymphocyte count; AMC, absolute monocyte count; ANC, absolute neutrophil count; Hgb, hemoglobin; MCV, mean corpuscular volume; WBC, white blood cell.

^∗^First available CBC in electronic medical record.

BM biopsy revealed a hypocellular marrow for age (40%), with evidence of dysmegakaryopoiesis and dysgranulopoiesis, but without fibrosis or an increased number of blasts. Megakaryocytes showed dysplastic features, predominantly exhibiting multinucleation with separated nuclei, while granulocytes were decreased in number, with dysplastic features including hypolobation and hypogranulation (see Figures 1 and 2). Erythroid elements were relatively increased in number, with maturation but no dysplastic features. Stage 1 hematogones were not detected. BM cytogenetic analysis identified a gain of 1q and an unbalanced translocation, der(1; 21) (q10; q10), in 35% (7/20) of metaphases. Flow cytometric analysis of this nonhemodilute BM aspirate was also abnormal, with an increase in mature lymphoid elements (64%) and a decrease in myeloid elements (18%, with 1% monocytes). The lymphocyte population was skewed toward the T‐cell lineage (97% CD3+ T‐cells), with decreased CD19+ B‐cells (2%) and 1% NK cells.

**FIGURE 1:**
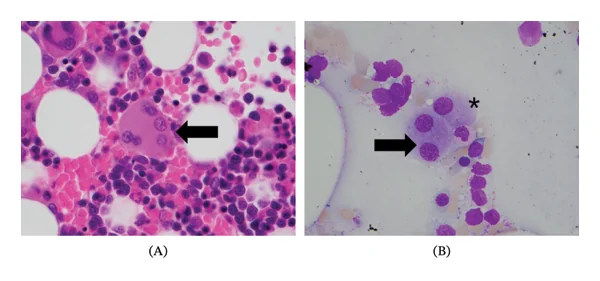
(A) H&E‐stained bone marrow clot section demonstrating a dysplastic megakaryocyte (arrow) with multiple separated nuclei characteristic of *GATA2* deficiency. (B) Giemsa‐stained bone marrow aspirate smear showing a dysplastic megakaryocyte (∗) with a unilobated nucleus, adjacent to another dysplastic megakaryocyte (arrow) with two separated nuclei.

**FIGURE 2:**
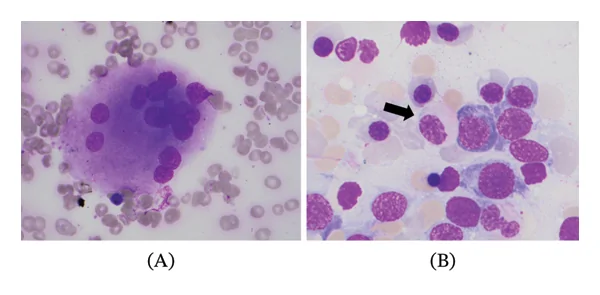
Giemsa‐stained bone marrow aspirate smears demonstrating (A) dysplastic megakaryocyte with multiple separated nuclei; (B) dysplastic myeloid cell (arrow) with a hypolobated nucleus.

Comprehensive myeloid next‐generation sequencing (NGS) panels of bone marrow aspirate, performed both in‐house and by a commercial laboratory, revealed no *GATA2* coding mutations but identified two known somatic truncating pathogenic mutations in the *STAG2* gene (c.2659A > T [variant allele frequency [VAF] 6%] [7]; c.3097C > T [VAF 13%]), both resulting in premature stop codons.

Peripheral blood flow cytometry demonstrated a relatively preserved T‐cell compartment, with CD3+ cells of 775/UL (RR: 741–2742). The absolute CD4+ T‐cell count was mildly reduced at 408/UL (RR: 413–1843), whereas the absolute CD8+ T cells, 349/UL (RR: 145–1422), and the CD4+/CD8+ ratio, 1.17 (RR: 0.44–5.78), were unaffected. B lymphocytes were markedly reduced, with CD19+ cells < 25/UL (RR: 53–722), while NK cells were absent at < 20/UL (RR: 30–675). Dendritic cells were not evaluated. Serum immunoglobulin levels were IgG 2937 mg/dL (RR: 791–1643), IgM 78 mg/dL (RR: 43–279 mg/dL), and IgA 189 mg/d (R: 66–436).

Given the strong suspicion for *GATA2* deficiency, a hereditary BM failure (BMF) syndrome panel was used to interrogate blood and skin fibroblast samples, with both detecting a previously described heterozygous pathogenic *GATA2* variant in the intron 4 (“+9.5Kb”) enhancer region (NM_032638.5: c.1017 + 572C > T) [6]. Based on the presence of MDS without increased blasts and the absence of “transformation mutations” (SETBP1, RAS pathway, and RUNX1), he was diagnosed with Spectrum 1 *GATA2* deficiency in the Largeaud et al. classification, intermediate between normal BM and overt leukemic transformation [8].

Indeed, over the next 5 months, his CBC showed worsening cytopenias, including development of moderate neutropenia and mild thrombocytopenia (Table 1), and repeat bone marrow now demonstrated a gain of 1q due to der(1; 21) (q10; q10) in 45% (9/20) of metaphases, consistent with clonal evolution.

He was thus referred for allo‐HSCT [9].

Because of the autosomal dominant germline *GATA2* deficiency and the risk of progression to AML, cascade genetic testing was instigated. There was no known family history of BMF syndromes on his maternal side. He has two maternal half‐sisters and five half‐brothers. The mother was negative for a *GATA2* mutation. Paternal history was largely unknown, with one half‐brother reported. Testing of the father remains pending at this point.

Rivaroxaban was held for three days prior to placement of a central venous catheter as access for his upcoming allo‐HSCT; however, he presented with acute right‐sided abdominal pain, with CT images showing a wedge‐shaped low‐attenuation region near the superior pole of the right kidney consistent with renal infarction. CT venogram of abdomen and duplex lower extremity studies did not reveal other new thromboses. He was started on therapeutic enoxaparin and later transitioned back to oral AC. In January 2026, he underwent allo‐HSCT from a 7/10 HLA‐mismatched unrelated donor following conditioning with fludarabine, busulfan, and total maarow irradiation along with posttransplant cyclophosphamide, engrafting successfully on Day 19+ (ANC 1.0; AMC 0.7). He was discharged on Day 25+ posttransplant on oral mycophenolate and tacrolimus as GVHD prophylaxis. The monocyte count corrected with engraftment, AMC 0.7 × 10^9^/L, increasing to 1.1, 2 months post‐HSCT. Chimerism studies on Days 30+, 60+, and 180+ posttransplant showed no detectable recipient‐derived CD3+ and CD33+ subsets, confirming complete donor‐derived hematopoiesis. To date, he has had no further thrombotic episodes while continuing therapeutic oral AC.

## 3. Conclusions

This case highlights a significant delay in the diagnosis of *GATA2* haploinsufficiency, despite a long history of monocytopenia, present for at least 12 years and an adolescent‐onset extensive DVT following minor surgery at age 16; the median age for presentation of *GATA2* deficiency [8]. The monocytopenia may have been present before age 11; however, prior hematologic data are unavailable. Notably, he also had a history of oral HPV at age 4. He subsequently developed mild anemia and lymphopenia at age 23, followed over a 6‐month period by mild thrombocytopenia and moderate neutropenia, reflecting progressive hypocellular MDS. As described by Largeaud et al., Spectrum 1 disease is characterized by MDS changes and clonal hematopoiesis, commonly with multiple *STAG2* loss‐of‐function mutations [8]. Delay in recognition of *GATA2* deficiency syndrome and intervention may allow for acquisition of additional high‐risk somatic mutations (SETBP1, RAS pathway, and RUNX1), rapidly leading to leukemic transformation (Spectrum 2 disease) [8]. Patients exhibiting diverse *GATA2* phenotypes may develop MDS or AML within 2 years [7].

Largeaud et al. noted [8] that somatic *STAG2* mutations are among the most frequently acquired genetic events in *GATA2* deficiency. In their cohort (*n* = 78), *STAG2* was highly enriched in Spectrum 1 disease compared to Spectrum 2, 47% vs. 18%. *STAG2*‐dependent chromatin accessibility guides *GATA1* binding in the erythroid and megakaryocytic lineages [10], and it has been suggested that, perhaps by altering chromatin accessibility of the *GATA2* cistrome, *STAG2* mutations are compensatory rather than leukemogenic, to increase *GATA2* expression [8]. In accordance, Kotmayer et al. found an increasing frequency of *STAG2* mutations with age in *GATA2*‐deficient patients (*n* = 218), and those with *STAG2* mutations had delayed onset of MDS, suggesting a potential protective role [11]. *STAG2* is X‐linked [7]; thus, our male patient has only one *STAG2* copy. The presence of two truncating *STAG2* mutations therefore suggests the existence of separate subclones. However, this cannot be definitively established without single‐cell sequencing or other phasing studies.

*GATA2* regulates key steps in endothelial cell specification, angiogenesis, vascular integrity, and lymphatic development; thus, *GATA2* deficiency may present with thrombosis at a young age, or primary lymphedema [4]. Clinical heterogeneity exists with respect to thrombotic manifestations in *GATA2* deficiency, with venous (especially catheter‐related) and arterial thrombosis occurring at atypical sites [4, 12]. Spinner et al. [4] reported thrombosis in 25% of patients (*n* = 57, median age 30), with half experiencing recurrent thrombotic events, including posttransplant, while arterial thrombotic complications occurred in 2 patients. Hofmann et al. [13] reported nine peri‐transplant thrombotic events (*n* = 15; median age 15.7), one arterial, 6 occurring posttransplant, while another cohort (*n* = 79; median age 24) of adult patients noted 3 venous and 3 arterial events [12]. It is hypothesized that the thrombotic risk remains high posttransplant, as the prothrombotic endothelial environment, related to impaired nitric oxide production, persists [4, 13, 14], despite transplantation. Hence, ongoing evaluation and a risk‐adapted management strategy should be pursued. In patients with a history of extensive thrombosis or those deemed to be at high risk of recurrent thrombosis, an individualized periprocedural AC strategy (e.g., bridging) should also be considered, after careful assessment of thrombotic and bleeding risks.

*GATA2* haploinsufficiency leads to immunodeficiency due to loss of a multilineage progenitor that gives rise to B lymphocytes, monocytes, NK cells, and dendritic cells, leading to cytopenias, predisposing patients to recurrent intracellular pathogens including viral infections (EBV and HPV); mycobacterium; fungal; and certain bacterial infections including *Pseudomonas*, *Klebsiella*, and rarely *Neisseria* [4]. HPV susceptibility is primarily due to dendritic cell and NK‐cell deficiency, with monocyte loss also contributing as they differentiate into tissue macrophages and can serve as antigen‐presenting cells. Our patient had excision of an oral papillomatous lesion at age 4, which was positive for HPV 6 and 11 genotypes. Recurrent chlamydial infections in *GATA2* haploinsufficiency have not been described to our knowledge; however, clearance of chlamydial infections requires both innate (macrophages, NK cells, and dendritic cells) [15] and adaptive immunity (B‐cell and T‐cell). His monocytopenia and low NK‐ and B‐cell numbers likely impaired both phagocytic function and adaptive immune responses, predisposing to recurrent intracellular infections. Urogenital structural abnormalities have also been described in *GATA2* deficiency [4, 16] and may increase susceptibility to urogenital infections; however, we found no evidence of abnormalities. Although recurrent chlamydial infections described here may therefore represent a manifestation of impaired host defenses due to *GATA2* deficiency, behavioral risk factors contributing to recurrence cannot be excluded; thus, a causal relationship would require confirmation in a larger cohort.

*GATA2* deficiency arises from over 150 mutations, involving both coding and noncoding regions [16, 17]. While coding region mutations, particularly missense variants, are most common, noncoding mutations, such as those involving the human +9.5 enhancer element within intron 4, have also been implicated (5%–10%) [7]. The c.1017 + 572C > T variant lies within the enhancer located 9.5Kb from the *GATA2* transcription start site, with the mutation specifically disrupting the Ets transcription binding motif of the enhancer, which controls *GATA2* transcription, resulting in haploinsufficiency of *GATA2* expression. Functional studies by Soukop et al. using murine models determined that this single‐nucleotide Ets‐binding mutation, though compatible with embryonic development, impairs hematopoietic stem cell maintenance and regeneration under conditions of hematopoietic stress [18]. Consistent with these observations, patients with Ets or those with +9.5Kb E‐box enhancer mutations (c.513‐540del28, c.532T > A) have a delayed phenotype onset compared to those harboring other mutation types, reflecting the partially preserved function of the enhancer variants, leading to development of the phenotype after cumulative hematopoietic stress, such as with repeated inflammation/infections [19].

Of note, commercial comprehensive myeloid NGS panels, such as those commonly employed to detect adult clonal hematopoiesis of indeterminate potential (‘CHIP’) or adult causes of cytopenias, generally rely on targeted myeloid gene exon sequencing (usually sequencing all exons and up to 10 bps at either end of introns); thus, they would not detect these noncoding region *GATA2* variants. As in our patient, a hereditary BMF panel that interrogates for known BMF gene mutations (or use of whole genome sequencing) is required to identify an intron 4 enhancer variant [6].

Cascade genetic testing is strongly recommended in family members, even if asymptomatic, as it identifies those at high risk of MDS/AML. Identified *GATA2* deficiency family members should have a baseline BM evaluation with cytogenetics and a somatic myeloid NGS panel for risk stratification, with consideration of preemptive allogeneic transplant in select cases, e.g., Spectrum 1 or 2 disease [7, 8]. Genetic testing is also critical to ensure that related hematopoietic stem cell donors do not harbor the pathogenic *GATA2* mutation [20].

In summary, this case highlights important diagnostic lessons in *GATA2* haploinsufficiency syndrome.

⁃ Persistent monocytopenia should always prompt consideration of *GATA2* deficiency, which is one of the most common genetic causes of MDS in patients presenting earlier than their 50^th^ birthday [21].
⁃ Severe (or in this case out‐of‐proportion) thrombosis with a negative thrombophilia workup in an otherwise healthy adolescent should prompt an extensive workup, which in the context of monocytopenia or recurrent infections (or other syndromic aspects) should include consideration of *GATA2* deficiency.
⁃ Negative myeloid sequencing panels that are primarily focused on coding mutations will not detect *GATA2* enhancer pathogenic variants.
⁃ Early recognition of *GATA2* deficiency syndrome enables timely genetic confirmation, cascade testing, and risk‐adapted strategies to prevent progression to AML, recurrent thrombosis, and infections.

## Author Contributions

Ahsan Wahab: conceptualization and writing–review and editing.

Moujtaba Zahweh: literature review and writing–review and editing.

Michael Stone: pathology expertise and writing–review and editing.

John G. Quigley: conceptualization and writing–review and editing.

## Funding

No funding was received for this study.

## Ethics Statement

Written informed consent was obtained from the patient for the publication of this case report and the accompanying images.

## Conflicts of Interest

The authors declare no conflicts of interest.

## Data Availability

Data sharing is not applicable to this article as no datasets were generated or analyzed during the current study.

## References

[1] Hsu A. P. , McReynolds L. J. , and Holland S. M. , *GATA2* Deficiency, Current Opinion in Allergy and Clinical Immunology. (2015) 15, no. 1, 104–109, 10.1097/aci.0000000000000126. PMC4342850 25397911

[2] Vinh D. C. , Patel S. Y. , Uzel G. et al., Autosomal Dominant and Sporadic Monocytopenia With Susceptibility to Mycobacteria, Fungi, Papillomaviruses, and Myelodysplasia, Blood. (2010) 115, no. 8, 1519–1529, 10.1182/blood-2009-03-208629. PMC2830758 20040766

[3] Wlodarski M. W. , Collin M. , and Horwitz M. S. , *GATA2* Deficiency and Related Myeloid Neoplasms, Seminars in Hematology. (2017) 54, no. 2, 81–86, 10.1053/j.seminhematol.2017.05.002. PMC5650112 28637621

[4] Spinner M. A. , Sanchez L. A. , Hsu A. P. et al., *GATA2* Deficiency: A Protean Disorder of Hematopoiesis, Lymphatics, and Immunity, Blood. (2014) 123, no. 6, 809–821, 10.1182/blood-2013-07-515528. PMC3916876 24227816

[5] Morimoto M. , Nishikawa T. , Hijikata A. et al., Case Report: *GATA2* Deficiency in Two Families With Novel Frameshift Variants Highlighting Phenotypic Diversity and Need for Early Diagnosis, Frontiers in Immunology. (2025) 16, 10.3389/fimmu.2025.1644552. PMC12350120 40821825

[6] Hsu A. P. , Johnson K. D. , Falcone E. L. et al., *GATA2* Haploinsufficiency Caused by Mutations in a Conserved Intronic Element Leads to MonoMAC Syndrome, Blood. (2013) 121, no. 19, 3830–3837, 10.1182/blood-2012-08-452763. PMC3650705 23502222

[7] Calvo K. R. and Hickstein D. D. , The Spectrum of *GATA2* Deficiency Syndrome, Blood. (2023) 141, no. 13, 1524–1532, 10.1182/blood.2022017764. PMC10082373 36455197

[8] Largeaud L. , Collin M. , Monselet N. et al., Somatic Genetic Alterations Predict Hematological Progression in *GATA2* Deficiency, Haematologica. (2023) 108, no. 6, 1515–1529, 10.3324/haematol.2022.282250. PMC10230419 36727400

[9] Rajput R. V. and Arnold D. E. , *GATA2* Deficiency: Predisposition to Myeloid Malignancy and Hematopoietic Cell Transplantation, Current Hematologic Malignancy Reports. (2023) 18, no. 4, 89–97, 10.1007/s11899-023-00695-7. 37247092

[10] Sudunagunta V. S. , Stewart E. M. , Chen Y. et al., *STAG2*-Dependent Chromatin Remodeling Enforces the Erythroid-Specific Gata1 Cistrome, Blood. (2026) 148, no. 6, 767–782. 10.1182/blood.2025030554 PMC13487485 42148659

[11] Kotmayer L. , Kozyra E. J. , Kang G. et al., Age-Dependent Phenotypic and Molecular Evolution of Pediatric MDS Arising From *GATA2* Deficiency, Blood Cancer Journal. (2025) 15, no. 1, 10.1038/s41408-025-01309-6. PMC12264048 40664679

[12] Donadieu J. , Lamant M. , Fieschi C. et al., Natural History of *GATA2* Deficiency in a Survey of 79 French and Belgian Patients, Haematologica. (2018) 103, no. 8, 1278–1287, 10.3324/haematol.2017.181909. PMC6068047 29724903

[13] Hofmann I. , Avagyan S. , Stetson A. et al., Comparison of Outcomes of Myeloablative Allogeneic Stem Cell Transplantation for Pediatric Patients With Bone Marrow Failure, Myelodysplastic Syndrome and Acute Myeloid Leukemia With and Without Germline *GATA2* Mutations, Biology of Blood and Marrow Transplantation. (2020) 26, no. 6, 1124–1130, 10.1016/j.bbmt.2020.02.015. PMC7276093 32088370

[14] Purgatorio G. , Piselli E. , Guglielmini G. et al., Germline *GATA2* Variant Disrupting Endothelial eNOS Function and Angiogenesis can be Restored by c-Jun/AP-1 Upregulation, Haematologica. (2022) 107, no. 5, 1072–1085, 10.3324/haematol.2021.278450. PMC9052902 34233447

[15] Roan N. R. and Starnbach M. N. , Immune-Mediated Control of Chlamydia Infection, Cellular Microbiology. (2008) 10, no. 1, 9–19. 10.1111/j.1462-5822.2007.01069.x 17979983

[16] Fabozzi F. , Mastronuzzi A. , Ceglie G. , Masetti R. , and Leardini D. , GATA 2 Deficiency: Focus on Immune System Impairment, Frontiers in Immunology. (2022) 13, 10.3389/fimmu.2022.865773. PMC9234111 35769478

[17] Wlodarski M. W. , Hirabayashi S. , Pastor V. et al., Prevalence, Clinical Characteristics, and Prognosis of *GATA2*-Related Myelodysplastic Syndromes in Children and Adolescents, Blood. (2016) 127, no. 11, 1387–1397, 10.1182/blood-2015-09-669937. 26702063

[18] Soukup A. A. , Zheng Y. , Mehta C. et al., Single-Nucleotide Human Disease Mutation Inactivates a Blood-Regenerative *GATA2* Enhancer, Journal of Clinical Investigation. (2019) 129, no. 3, 1180–1192, 10.1172/jci122694. PMC6391105 30620726

[19] Hsu A. P. , Paul S. , Kwan J. L. et al., *GATA2* at 14: Genotype-Phenotype Correlations, Haematologica. (2026) 10.3324/haematol.2024.287013. PMC13725280 41537337

[20] Sakata N. , Okano M. , Masako R. et al., Donor-Derived Myelodysplastic Syndrome After Allogeneic Stem Cell Transplantation in a Family With Germline *GATA2* Mutation, International Journal of Hematology. (2021) 113, no. 2, 290–296, 10.1007/s12185-020-02980-0. 32865708

[21] Cazzola M. , Myelodysplastic Syndromes, New England Journal of Medicine. (2020) 383, no. 14, 1358–1374, 10.1056/nejmra1904794. 32997910

